# A role for WDR5 in TRA-1/Gli mediated transcriptional control of the sperm/oocyte switch in *C. elegans*

**DOI:** 10.1093/nar/gku221

**Published:** 2014-03-20

**Authors:** Tengguo Li, William G. Kelly

**Affiliations:** Biology Department, Emory University, Atlanta, GA 30322, USA

## Abstract

The hermaphrodite germline of *Caenorhabditis elegans* initially engages in spermatogenesis and then switches to oogenesis during late stages of larval development. TRA-1, a member of the Ci/Gli family of transcriptional repressors, plays an essential role in this switch by repressing genes that promote spermatogenesis. WDR5 proteins are conserved components of histone methyltransferase complexes normally associated with gene activation. However, two *C. elegans* WDR5 homologs, *wdr-5.1* and *wdr-5.2* are redundantly required for normal TRA-1 dependent repression, and this function is independent of their roles in histone methylation. Animals lacking *wdr-5.1/wdr-5.2* function fail to switch to oogenesis at 25°C, resulting in a masculinization of germline (Mog) phenotype. The Mog phenotype is caused by ectopic expression of *fog-3*, a direct target of TRA-1 repression. WDR-5.1 associates with the *fog-3* promoter and is required for TRA-1 to bind to *fog-3* promoter. Other direct targets of TRA-1 are similarly derepressed in the double mutant. These results show that WDR5 plays a novel and important role in stabilizing transcriptional repression during *C. elegans* sex determination, and provide evidence that this important protein may operate independently of its established role in histone methyltransferase complexes.

## INTRODUCTION

Sex determination in the nematode *Caenorhabditis elegans* is genetically controlled by a complex negative regulatory pathway that is ultimately linked to the sex chromosome/autosome ratio (1). XX embryos (2X:2A) undergo hermaphrodite development and XO animals (1X:2A) develop as males (2,3). The hermaphrodite is somatically female, but the first XX germ cells completing meiosis in larval stages enter spermatogenesis. After the final larval molt XX germ cell development switches to oogenesis, and oocytes in the adult are fertilized by the sperm produced in the preceding larval stage to yield self-progeny. The X:A ratio provides differing complementary doses of several X- and autosome-linked regulatory factors that ultimately control the expression of the master regulator *xol-1*, which regulates a cascade of negative genetic interactions that control sex determination and/or dosage compensation (1). At the terminus of the sex determination cascade is TRA-1, a Zinc finger transcription factor, which plays a global role in determining the sexual identity of both germline and soma (4). The TRA-1A protein contains five Zinc fingers and is closely related to the *Drosophila Cubitus interruptus* (Ci) and mammalian Gli transcriptional repressors (5,6). Ci/Gli proteins play pivotal roles in development, stem cell maintenance and tumorigenesis as transducers of Hedgehog signaling (7). Although there is no known Hedgehog signaling pathway in *C. elegans*, it is proposed that the worm sex determination pathway was adapted or derived from the Hedgehog pathway (2).

TRA-1 promotes female development by inhibiting genes that drive male differentiation (4,5). In germ cells, TRA-1 promotes the switch to oogenesis by inhibiting two genes, *fog-1* and *fog-3*, which are required for spermatogenesis (1). TRA-1 binds to the promoter of *fog-3* and directly represses its transcription (8). In somatic tissues, two sexual development genes, *mab-3* and *egl-1*, have also been identified as direct targets of TRA-1 repression (8–10). Like Ci/Gli, TRA-1 undergoes proteolytic processing to generate a 90 kDa C-terminal truncated isoform. Post-transcriptional modifications of this truncated protein generate multiple isoforms with molecular weights ranging from 90 to 110 kDa (11). Importantly, these isoforms have feminization activity and appear to be the active forms (11).

Precisely, how TRA-1 regulation contributes to its regulation of sex determination is not fully understood. Schvarzstein and Spence reported that the overall protein level of TRA-1 was higher in hermaphrodites, indicating that protein levels are important for TRA-1 regulation (11). In contrast, Segal *et al.* found that TRA-1 protein levels were similar in both males and hermaphrodites, but that more TRA-1 was localized in nuclei of hermaphrodites than in males, suggesting that TRA-1 localization plays an important role in regulating sex determination (12). Regulation of TRA-1 levels, however, is clearly important. Immediately upstream of *tra-1* in the sex determination genetic pathway are the *fem* genes (*fem-1*, *fem-2* and *fem-3*), which negatively regulate *tra-1*. The FEM proteins (FEM-1, FEM-2 and FEM-3) are members of the CUL-2-based ubiquitin ligase complex, and TRA-1 regulation involves CUL-2 mediated, ubiquitin-directed proteasome targeting and degradation (13).

Regulation of TRA-1's association with, or access to, its target loci are other possible modes of regulation. We previously showed that mutations in the conserved histone methyltransferase complex component, *wdr-5.1*, cause a low penetrance masculinization of germline (Mog) phenotype in hermaphrodites (14), suggesting that *wdr-5.1* and/or histone methylation play a role in germline sex determination. WDR5 is a core subunit of the highly conserved Mixed-Lineage Leukemia (MLL) and Set/COMPASS (Complex Proteins Associated with Set1) histone H3 lysine 4 (H3K4) methyltransferase complexes (HMT), hereafter referred to as Set/MLL complexes (15). H3K4 methylation is generally associated with transcription competency and/or activity (16), and WDR5 has been observed to be essential for Set/MLL complex activities in all organisms tested (14,17–19). Loss of WDR5 in mammalian cells results in global decreases of H3K4 mono-, di-, and trimethylation (H3K4me1/2/3) to various degrees (17). Knockdown of WDR5 leads to reduction of mono- and trimethylation levels and a variety of phenotypes, including somatic and gut defects, in *Xenopus* *laevis* (18). In *C. elegans*, deficiencies of *wdr-5.1* or *ash2*, another conserved member of Set/MLL complexes, induce worm lifespan extension, and this is proposed to be linked to their functions in germ cells (20). Biochemical studies in yeast and mammalian cells demonstrate that WDR5 along with two other conserved proteins, Ash2L and RbBP-5, form a core complex that is essential for complex stability and enzymatic activity (17,21). Structure studies reveal that WDR5 recognizes the Ala1, Arg2 and Thr3 of histone H3, and binds to H3 regardless of the methylation status of Lys4 (22–25).

A conserved WDR5-interacting (Win) motif located in the N-SET region of the histone methyltransferase MLL1 was recently identified (26). Strikingly, the Win sequence in MLL1 shares homology with the N-terminus of histone H3, and indeed the Win peptide binds to the same site with which WDR5 binding to histone H3 occurs. This finding suggests that WDR5's interactions with MLL and histone H3 may be mutually exclusive, and thus WDR5's ability to interact with nucleosomes may not always directly relate to its function in Set/MLL complexes. WDR5 has been reported to also function within complexes without HMT activity. Garapaty *et al.* reported that WDR5 was associated with the nuclear receptor complex interacting factor-1 (NIF-1) to promote the expression of target genes (27). WDR5 is also reported to be a subunit of CHD8 containing ATP-dependent chromatin remodeling complexes and the histone acetyltransferase complexes ATAC and MOF (28). None of these complexes are known to contain Set1- or MLL-related histone methyltransferase activity. A recent report also demonstrated a role for WDR5 in stem cell self-renewal that involves interactions with the pluripotency factor Oct4. Interestingly, WDR5 interactions with Oct4 were observed in complexes in which other conserved Set/MLL complex members (e.g. Rbbp5 and Ash2l) were not detected (29).

We and others recently reported that conserved Set/MLL complex components, including a worm ortholog of WDR5, WDR-5.1, are required to maintain H3K4 methylation in early embryos and in adult germline stem cells, and that the loss of *wdr-5.1* causes defects in germline stem cell maintenance and proper germline development (14,30). In our study, we observed that among the three *C. elegans* WDR5 homologs, loss of just one of them, WDR-5.1, caused a completely penetrant defect in H3K4 methylation. Interestingly, although loss of the two other *wdr-5* homologs, *wdr-5.2* or *wdr-5.3*, created no observed defects in H3K4 methylation, *wdr-5.1*;*wdr-5.2* double mutants exhibited more severe germline developmental defects than either single mutant. The absence of H3K4 methylation defects in the *wdr-5.2* single mutant and the synthetic germ cell defects with *wdr-5.1* suggested a redundancy in function for these two genes that is separate from WDR-5.1's role in H3K4 methylation. In these studies, we further investigate this additional role for WDR5 in germ cell development, and show that *wdr-5.1* and *wdr-5.2* are redundantly required for the switch from spermatogenesis to oogenesis in hermaphrodites to occur normally. The two *C. elegans wdr-5* homologs are redundantly required for TRA-1-dependent repression of *fog-3* in adult germ cells; the loss of both results in a Mog phenotype. TRA-1, the transcriptional repressor of *fog-3*, is abnormally depleted from the nuclei of the adult germ cells in *wdr-5.1;wdr-5.2* double mutants, indicating that TRA-1's stable nuclear localization, presumably involving its association with its target loci, is dependent on WDR5 function. We conclude that TRA-1 mediated *fog-3* repression in adult germline is dependent on WDR5. This indicates that WDR5, in addition to its roles in stabilizing histone methyltransferase complexes, has additional roles that can include stabilizing transcriptional repressor complexes at target loci.

## MATERIALS AND METHODS

### Worm strains

*Caenorhabditis elegans* strains were maintained using standard conditions at 20°C unless otherwise noted. N2 (Bristol) was used as the wild-type (WT) strain. The following mutant strains were used in this study: *set-2(tm1630)III*, *wdr-5.1(ok1417)III*, *wdr-5.2(ok1444)X*, *rbbp-5(tm3463)II*, *tra-1(e1834)III*. The *wdr-5.1;wdr-5.2* double mutant strain was generated by cross between *wdr-5.1(ok1417)III* and *wdr-5.2(ok1444)X.* The genotypes were confirmed with polymerase chain reaction (PCR). The *wdr-5.1::GFP* integrated transgenic strain was a generous gift from Dr F. Palladino, Ecole Normale Superieure de Lyon, France. The *wdr-5.1;wdr-5.2* strain carrying *wdr-5.1::GFP* was generated through crossing and the genotypes were confirmed by PCR. Primers for genotyping are shown in Supplementary Table S1. The GFP-fused *daf-16* strain, *daf-2;RX87: daf-2(e1370);daf-16(mgDf47);daf-16::gfp*, was a kind gift from Dr Sylvia Lee, Cornell University.

### RNAi analysis

Double stranded RNA (dsRNA) corresponding to *wdr-5.1*, *wdr-5.3*, *rbbp-5*, *fog-3*, *fem-3* and *fog-1* were generated using the Ribomax Large Scale RNA production kit (Promega). L4 staged *wdr-5.1;wdr-5.2* animals were soaked in 1 μg/μl of dsRNA for 24 h at 20°C. The worms were then grown on RNAi feeding plates with bacteria expressing dsRNA targeting the corresponding gene, or carrying the empty L4440 vector for control experiments. After the first 24 h, the worms (P0) were transferred to a new set of feeding plates. F1 animals (24 h post L4) were assessed for sterility and other phenotypes and/or analyzed by immunofluorescence.

### Immunofluorescence

Worms were dissected and processed as described before (14,31) and treated with respective primary antibody overnight and secondary antibody for 4 h at room temperature. Rabbit anti-H3K4me3 (1:1000) was purchased from Abcam (ab8580). Mouse monoclonal antibody against H3K4me2 (CMA303; 1:20) were gifts from Dr. Hiroshi Kimura (Osaka University, Japan). Mouse monoclonal antibody against GFP (1:500) was purchased from Millipore. Rabbit anti-TRA-1 (1:100) antibodies were generous gifts from Drs. David Zarkower (University of Minnesota) and Andrew Spence (University Toronto). 4',6-Diamidino-2-Phenylindole (DAPI) (Sigma, 2 μg/μl) was used to counter-stain deoxyribonucleic acid (DNA). All secondary antibodies were purchased from Molecular Probes and were used at 1:500 dilutions: goat anti-mouse IgG (Alexafluor 488); goat anti-rabbit IgG (Alexafluor 594), donkey anti-rabbit IgG (Alexafluor 488); donkey anti-mouse IgG (Alexafluor 594). Worms were mounted in anti-fade reagent (Prolong Gold, Molecular Probes). Images were collected using a Leica DMRXA fluorescence microscope and analyzed with Simple PCI software (Hamamatsu Photonics).

### Quantitative RT-PCR analysis

RNA was purified from dissected gonads or whole worm. Approximately eighty gonads were dissected out in 30 μl of dissection buffer (0.5 μM levamisole and 0.01% Tween-20 in 1× egg buffer) and transferred into a depression glass well slide containing 200 μl of wash buffer (0.01% Tween-20 in 1× egg buffer) using a mouth pipette. After washing, the gonads were transferred to Eppendorf tubes and centrifuged briefly to remove excess liquid. One hundred microliters of Trizol was added and the samples were subsequently frozen in liquid nitrogen. To purify RNA from whole worms, 100 L4 or adults were washed and transferred to 20 μl of 1× phosphate buffered saline (PBS). Two hundred microliters Trizol reagent was added to the samples that were then frozen in liquid nitrogen. The samples were thawed, vortexed and refrozen four times. After four cycles, the sample was extracted and precipitated with isopropanol, washed with 75% ethanol, and dissolved in RNase-free water. The isolated RNA was reverse-transcribed using the Invitrogen First Strand Synthesis System with Oligo(dT) 20 priming. The results were normalized to *actin-1* (*act-1*). The qPCR analyses were performed using a real-time PCR instrument (BIO-RAD CFX 96 real time system) with reaction mix (iQ SYBR Green Master Mix; Bio-Rad). The qPCR primers are shown in Supplementary Table S1.

### Western blot analysis

For western blot analysis of TRA-1 protein in hermaphrodite germ cells, 160 gonads were dissected from adults and washed with PBS as described above. After removing extra PBS, sodium dodecyl sulphate-polyacrylamide gel electrophoresis (SDS-PAGE) sample buffer were added. For analysis of *tra-1(e1834)*, whole adult worms were picked and transferred to 10 μl of PBS in an Eppendorf tube. Ten microliters of 2× SDS-PAGE sample buffer was added to the samples and boiled for 5 min. The samples were loaded and run on an 8% SDS-PAGE gel and transferred to PVDF membranes. The proteins were probed with a rabbit polyclonal antibody against TRA-1 at 1:1000 (a gift from Dr Zarkower, University of Minnesota) or anti-ACTIN antibody (Chemicon) at 1:1000.

### Chromatin IP (ChIP) assays

The H3K36me3 and H3K4me3/2 ChIP were done as described by (32). Briefly, for ChIP assay for H3K4me3/2, H3K36me3 and H3, worms were collected in M9 buffer and bleached in 1N NaOH/20% bleach to collect embryos. The embryos were hatched overnight at 20°C and the synchronized L1 were transferred to Nematode Growth Medium (NGM) plates and grown at 25°C. L4 or adults were collected into 1× PBS containing proteinase inhibitor (Roche, Cat. no.: 11836170001) and frozen in liquid nitrogen. Ground frozen worm powder was fixed in 1% formaldehyde and sonicated for 80 s (4 s on/10 s off) at 20% in a Fisher Scientific Model 500 Sonic Dismembrator. For anti-GFP ChIP, *wdr-5.1;wdr-5.2;wdr-5.1::gfp* animals were grown at 25°C. Synchronized adult worms were collected for ChIP. As a GFP ChIP positive control, synchronized adult *daf-2;RX87: daf-2(e1370);daf-16(mgDf47);daf-16::gfp* animals were shifted to 25°C for 2 h before collecting for ChIP (33). Immunoprecipitation was done using GFP-TRAP_M (Chromotek). For TRA-1 ChIP, worms were ground in liquid nitrogen and fixed in 1% paraformaldehyde for 20 min at room temperature and sonicated for 80 s. Rabbit anti-H3K4me3 (Abcam, ab8580), anti-H3K36me3 (ab9050) and anti-H3 (ab1791), rabbit anti-TRA-1 (a gift from Dr Andrew Spence) was used for immunoprecipitation. The rest of the ChIP procedure was done using a ChIP assay kit (Millipore, Cat. no.: 17-295). The primers (*fog-3*-F/R-promoter for H3K3me3 and *fog-3*-F2/R2 for H3K36me3) for qPCR are shown in Supplementary Table S1 and primers for *sod-3* was based on (33).

## RESULTS

### Fertility defects in *wdr-5.1;wdr-5.2* double mutants

The *C. elegans* genome contains three homologs of WDR5: *wdr-5.1*, *wdr-5.2* and *wdr-5.3* (Supplementary Figure S1). *wdr-5.1* shares the greatest homology with human WDR5 (70%), and *wdr-5.2* and *wdr-5.3* share 65 and 52% homology, respectively, with *wrd-5.1*. *wdr-5.1(ok1417)* mutant animals have low embryo lethality (Emb; 12%) and sterility (Ste; 2.5%) at 20°C and these phenotypes rise to 36 and 29%, respectively at 25°C (Table 1 and (14)). At 25°C, this mutant also exhibits a temperature-sensitive ‘mortal germline’ phenotype, in which the fraction of sterile progeny increases with each successive generation (14,34). However, the *wdr-5.2 (ok1444)* deletion mutant does not exhibit obvious phenotypes at either 20 or 25°C (Table 1). Surprisingly, *wdr-5.1;wdr-5.2* double mutants exhibit significantly more embryonic lethality and sterility (19 and 16%, respectively) at 20°C. Notably, if shifted to 25°C during the L4 larval stage, *wdr-5.1;wdr-5.2* double mutants develop into adults with major defects in reproduction (an average brood size of 35 compared to 214 in WT), and 42% of their progeny die as embryos, which is similar to that of *wdr-5.1* (36%). The remainder hatch and develop into adults, but 100% of these animals are sterile (Table 1).

**Table 1.** Phenotypic analysis of *wdr-5* mutants

	Emb		Sterility	
	20°C	25°C	20°C	25°C
WT^a^	0.6% (*n* = 619)	2.7% (*n* = 1509)	0 (*n* = 114)	0.75% (*n* = 133)
*wdr-5.1(ok1417)*^a^	12% (*n* = 1201)	36% (*n* = 557)	2.5% (*n* = 119)	29% (*n* = 89)
*wdr-5.2(ok1444)*	0 (*n* = 693)	1.7% (*n* = 1320)	0 (*n* = 101)	0.9% (*n* = 111)
*wdr-5.1;wdr-5.2*	19% (*n* = 616)	42% (*n* = 352)	16% (*n* = 141)	100% (*n* = 99)

^a^Data from Li and Kelly (14).

Adult animals were picked and shifted to 25°C. The next generation (F1) was checked for sterility. Animals containing no embryos were designated sterile. Embryos that were laid were counted and those that failed to hatch after 24 h were scored as embryonic lethal (Emb).

**Figure 1.**
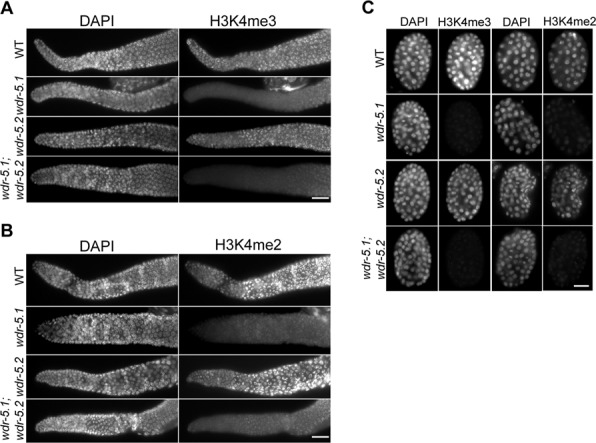
Loss of WDR-5.2 has no obvious effect on H3K4 methylation. Dissected and fixed gonads or embryos from wild-type (WT), *wdr-5.1(ok1417)*, *wdr-5.2(ok1444)* or *wdr-5.1;wdr-5.2* double mutants were probed with rabbit anti-H3K4me3 or mouse anti-H3K4me2 antibodies; DNA was counterstained with DAPI. Exposure times were the same for each condition. Gonads are displayed with the distal region to the left. (**A** and **B**) In contrast to *wdr-5.1* mutants, which show dramatic reduction of H3K4me3 (panel A) and H3K4me2 (panel B), *wdr-5.2* single mutants exhibit normal levels of H3K4me2/3. *wdr-5.1;wdr-5.2* double mutant exhibited pattern same as that in *wdr-5.1* single mutants. Scale bars = 20 μm. (**C**) *wdr-5.1* is essential for normal maintenance of H3K4me3 and H3K4me2 in embryos while *wdr-5.2* is largely dispensable. Scale bar = 10 μm.

The severe phenotypes are intriguing since they seem to be separate from H3K4me defects, which are caused by the loss of WDR-5.1 alone. We previously showed that WDR-5.1, together with another conserved Set/MLL component, RBBP-5, is required for the normal maintenance di- and trimethylation of H3K4 in early embryos and in the stem cell pool of the postembryonic germline (14). In contrast, neither individual deletion of *wdr-5.2 (ok1444)*, nor knockdown of *wdr-5.3* by RNAi, results in detectable H3K4me2/3 defects in either early embryos or adult germ cells at 20 or 25°C (Figure 1; (14) and data not shown) . Furthermore, *wdr-5.1*;*wdr-5.2* double mutants and triple mutant (RNAi knockdown of *wdr-5.3* in *wdr-5.1;wdr-5.2*) exhibit defects in H3K4 methylation that are identical to those observed in *wdr-5.1* single mutants (Figure 1 and data not shown), indicating that WDR-5.2 and WDR-5.3 are dispensable for H3K4 methylation. Taken together, these results suggested that *wdr-5.1* and *wdr-5.2* have overlapping functions that are essential for normal fertility at elevated temperatures, and that these functions might be independent of WDR-5.1's role in the maintenance of global H3K4 methylation.

### *wdr-5.1;wdr-5.2* double mutants exhibit temperature sensitive sperm/oocyte switch defects

**Figure 2.**
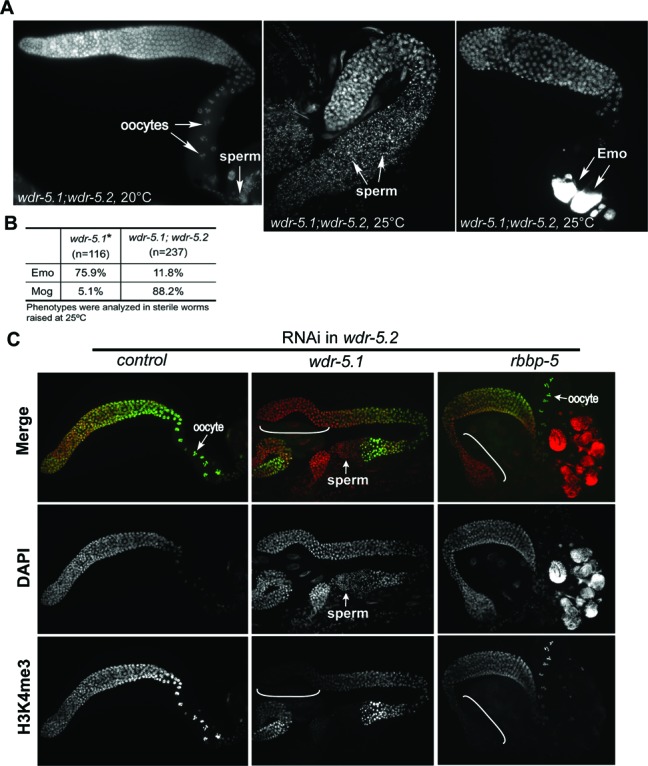
*wdr-5.1;wdr-5.2* double mutants exhibit a highly penetrant, temperature sensitive Mog phenotype. (**A**) DAPI staining of extruded and fixed gonads from *wdr-5.1;wdr-5.2* double mutants. Young adults were raised at 20°C or shifted to 25°C and gonads from their F1 adult progeny (24 h post L4) were stained with DAPI. *wdr-5.1;wdr-5.2* double mutants raised at 20°C show normal germ cell development and fertility (left panel), but have high Mog (sperm only; center panel), and low Emo (highly polyploid oocytes; right panel) phenotype frequencies at 25°C. (**B**) Comparison of the frequency of Mog and Emo phenotypes in sterile animals of single *wdr-5.1(ok1417)* and double *wdr-5.1;wdr-5.2* mutants raised at 25°C. *Data from (14). (**C**) *wdr-5.1* or *rbbp-5* were knocked down by RNAi in *wdr-5.2 (ok1444)* mutant animals. The F1 progeny of treated animals were grown at 25°C. Gonads dissected from animals 24 h post L4 were stained with antibody against H3K4me3 (green) and counter stained with DAPI (red).

To further characterize the sterility phenotype of *wdr-5.1;wdr-5.2* double mutants, we dissected the gonads from hermaphrodites and stained the DNA with DAPI. As shown in Figure 2A, at 20°C, the fertile *wdr-5.1;wdr-5.2* double mutant hermaphrodites have normal, well-developed and organized gonads. Germ cells first undergo spermatogenesis and later switch to oogenesis, so both sperm and oocytes are present in the adult gonad. However, 88.2% of the gonads dissected from the sterile *wdr-5.1;wdr-5.2* worms grown at 25°C contained only sperm (Figure 2A middle panel and B). This Mog phenotype (masculinization of germline) is indicative of defective transition from spermatogenesis to oogenesis. The germ cells in the remaining gonads (11.8%) had successfully switched to oogenesis, but the oocytes showed an endoreplication, or endomitotic (Emo) phenotype (Figure 2A right panel and B). The sterility of the double mutants was specific to the loss of WDR5 function, since normal fertility was restored by a transgene expressing only WDR-5.1::GFP (Supplementary Figure S2). These results indicate that WDR-5.1 and WDR-5.2's roles in germline sex regulation are redundant.

In contrast to the double mutant, the majority (75.9%) of the sterile animals from *wdr-5.1* single mutants raised at 25°C are Emo and only 5.1% are Mog ((14) and Figure 2B). The difference in the penetrance of the Mog and Emo phenotypes at 25°C, depending on the presence or absence of WDR-5.2, is curious as neither phenotype is observed at any significant frequency in the *wdr-5.2* single mutant. One explanation is that WDR-5.2 is dispensable for germline sex transitions in the presence of WDR-5.1, and may play a compensatory role in the absence of WDR-5.1. Consistent with this, *wdr-5.2* mRNA levels are similar in both WT and *wdr-5.1* at 20°C, but *wdr-5* is upregulated 3-fold in *wdr-5.1* at 25°C (Supplementary Figure S3). Taken together, these results suggest that WDR-5.2 is redundant with WRD-5.1 during germline sex transitions, but WDR-5.1's role in oocyte development, as in H3K4 methylation, is independent of WDR-5.2. Thus, the Emo phenotype is more closely linked to the H3K4 methylation defects observed in *wdr-5.1* single mutants. It is important to note that animals with mutations in another Set/MLL complex component, *rbbp-5*, show robust H3K4 methylation defects in adult germ cells and also display a strong Emo defect (14). Importantly, *rbbp-5* mutants are not Mog at either 20 or 25°C, further suggesting that the role of WDR-5.1/.2 that underlies the Mog phenotype is independent of its role in Set/MLL complex activities.

To further test this, we knocked down *wdr-5.1* or *rbbp-5* in *wdr-5.2(ok1444)*, respectively. Gonads were dissected from F1 animals grown at 25°C and stained with antibodies against H3K4me3. As shown in Figure 2C, H3K4me3 was efficiently knocked down to an undetectable level by RNAi of either *wdr-5.1* or *rbbp-5* in the distal germ cells, as previously reported (14). RNAi of *wdr-5.1* in *wdr-5.2* also resulted in the Mog phenotype. In contrast, *rbbp-5(RNAi)* in *wdr-5.2(ok1444)* did not produce a Mog phenotype: oogenesis occurred and Emo phenotype was observed in *rbbp-5* RNAi worms (Figure 2C). To test if the Mog phenotype resulting from *wdr-5.1* RNAi is specific to *wdr-5.1*, we analyzed the specificity of RNAi by qRT-PCR. As shown in Supplementary Figure S4, RNAi knockdown of *wdr-5.1* in N2 led to an 80% decrease in *wdr-5.1* mRNA, while the mRNA levels of *wdr-5.2* and *wdr-5.3* were not affected. There was no increase in *wdr-5.*2 observed in this experiment as RNAi was performed at 20°C (Figure S3). Together these data indicate that both WDR-5.1 and WDR-5.2 are redundantly required for the normal switch from spermatogenesis to oogenesis, and this requirement is distinct from the Set/MLL roles of WDR-5.1 and RBBP-5 in H3K4 methylation. It furthermore suggests that proper regulation of H3K4 methylation is important for normal oocyte development in *C. elegans*.

### Normal regulation of *fog-3* and *fog-1* require *wdr-5.1/wdr-5.2* function

**Figure 3.**
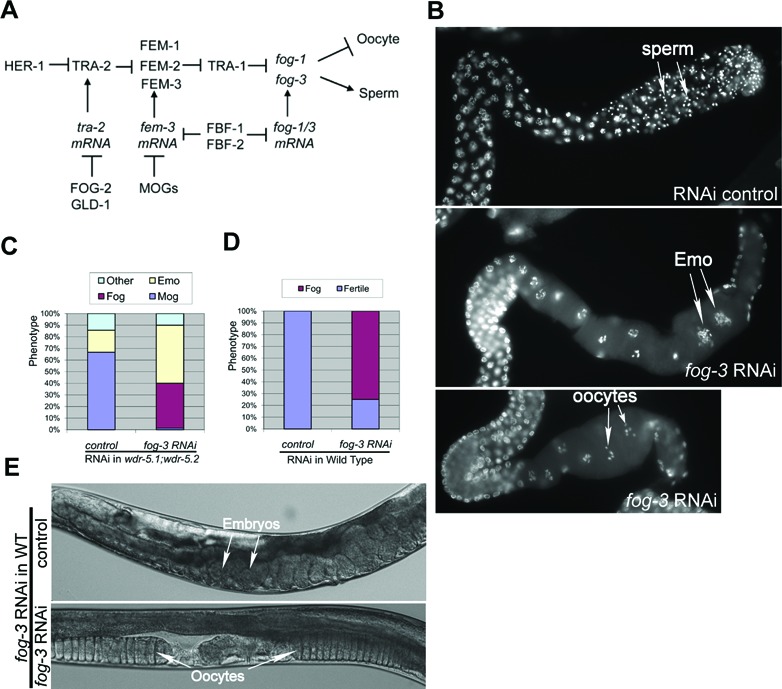
Knockdown of *fog-3* rescues the Mog phenotype in *wdr-5.1;wdr-5.2* double mutants. (**A**) Diagram of genetic pathways of germline sex determination in *Caenorhabditis elegans*. Barred lines indicate negative regulation and arrows indicate positive regulation. Figure is modified from (1). (**B**) RNAi inactivation of *fog-3* in *wdr-5.1;wdr-5.2* converts the Mog phenotype to a feminized germline phenotype (Fog). DAPI-stained double mutant gonads are displayed with the proximal oogenesis region to the right. RNAi knockdown of *fog-3* in *wdr-5.1;wdr-5.2* converts the Mog phenotype (RNAi control, upper panel) to germ cells that enter oogenesis (lower panels). *fog-3(RNAi)* in the double mutants also causes an Emo phenotype, which is not observed in wild-type *fog-3(RNAi)* animals (middle panel). (**C**) Quantitative summary of the phenotypes of *fog-3* RNAi treated *wdr-5.1;wdr-5.2*, +/− *fog-3(RNAi).* (**D**) Quantitative summary of the phenotypes of *fog-3* RNAi treated wild-type (N2), +/− *fog-3(RNAi)*. (**E**) DIC images show the Fog phenotype in N2 animals treated with *fog-3* RNAi.

Germline sex determination in *C. elegans* is controlled by a pathway composed of a series of sequential activities that function as genetic switches (Figure 3A, for review, see (1)). Disruption of any gene in the pathway can lead to defective transition from spermatogenesis to oogenesis and/or other germline defects. As discussed above, *wdr-5.1* and *wdr-5.2* are required for the normal switch to oogenesis, particularly during growth at elevated temperatures. The genes *fog-1* and *fog-3* play a decisive role in promoting spermatogenesis. *fog-3* loss-of-function (*lf*) mutant hermaphrodites fail to make sperm and only produce oocytes, a phenotype called Fog (feminization of germline). To investigate the genetic interaction between *fog-1/3* and *wdr-5*, we knocked down *fog-1* or *fog-3* in *wdr-5.1;wdr-5.2* double mutants by RNAi. RNAi knockdown of *fog-1* failed to consistently reverse the Mog phenotype in *wdr-5.1;wdr-5.2* double mutants, possibly due to RNAi inefficiencies in targeting this gene. In contrast, we observed a near complete reversal of the Mog phenotype to Fog in the *wdr-5–1;wdr5.2* double mutants treated with *fog-3(RNAi)*. As shown in Figure 3B and C, 66.7% of the dissected gonads of *wdr-5.1;wdr-5.2* RNAi control animals displayed the Mog phenotype. In striking contrast, 89% of the gonads of *wdr-5.1;wdr-5.2; fog-3(RNAi)* animals produced only oocytes. Interestingly, 39% of the Fog animals had gonads with normal oocyte morphology (Figure 3B bottom panel), whereas the rest also displayed an Emo phenotype (Figure 3B, middle panel). In a parallel experiment, RNAi knockdown of *fog-3* in WT worms resulted in 75% sterility, and the sterile animals showed the Fog phenotype (Figure 3D and E). The Emo phenotype was very rarely observed in *fog-3(RNAi)* in WT animals (2–3%; not shown) which further uncouples this phenotype from the defect in sperm/oocyte transition in our assays. The Emo gonads in the Fog animals is likely a compound phenotype, i.e. defective H3K4 methylation (loss of WDR-5.1 activity) in the context of the *fog-3(RNAi)*.

### *fog-3* transcriptional repression is defective in *wdr-5.1;wdr-5.2* germ cells

**Figure 4.**
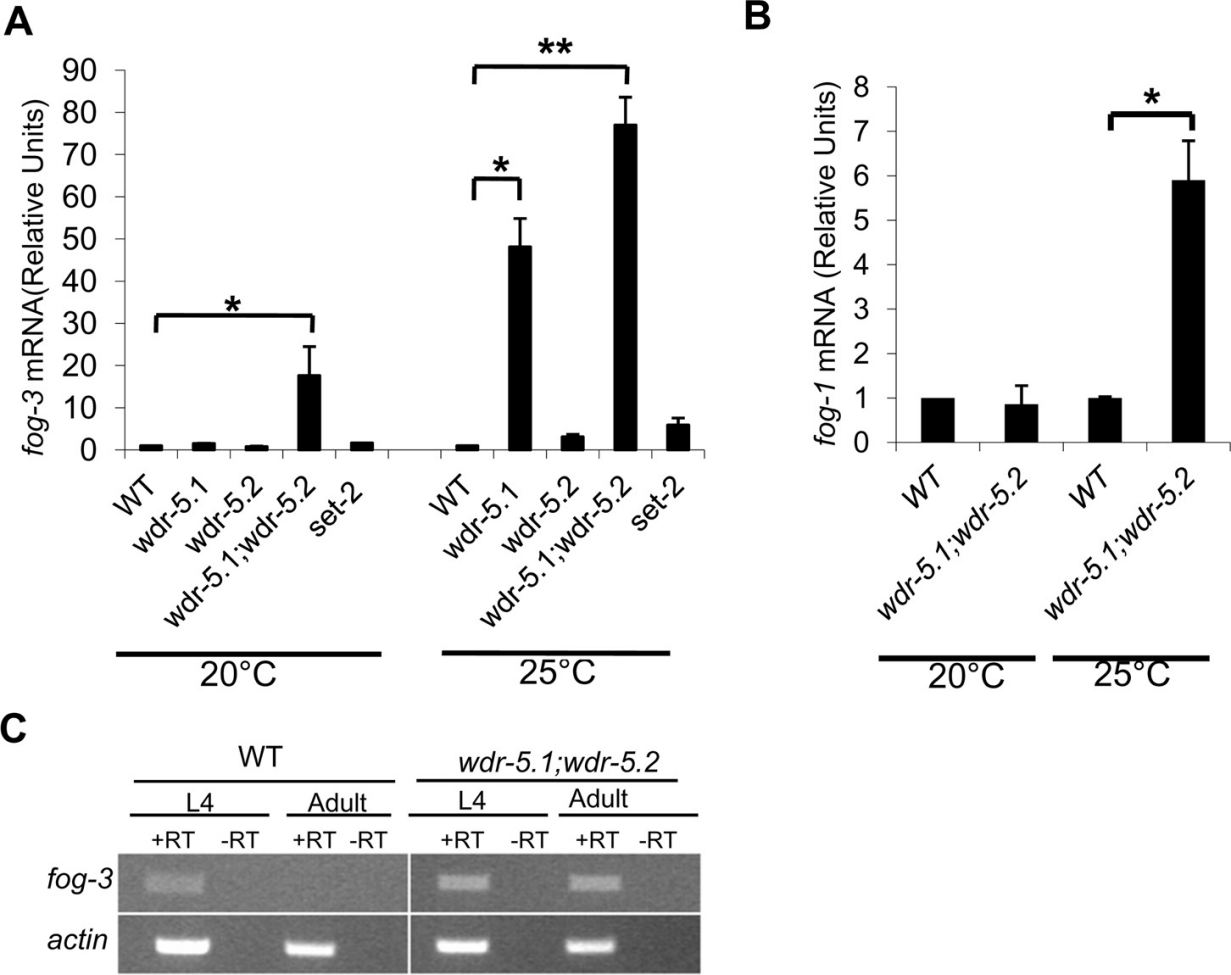
WDR5 is required for *fog-3* and *fog-1* repression in adult hermaphrodites. RNA was purified from dissected gonads and RNA levels were quantified by qRT-PCR using *fog-3* or *fog-1* specific primers. The actin gene *act-1* was used as an internal control. Signals were normalized to WT *fog-3* RNA levels, which were set to equal 1.0. Error bars represent SEM from two independent experiments. *P < 0.05 and **P < 0.01; Student's *t-*test. (**A**) qRT-PCR analysis of *fog-3* mRNA in different mutants. (**B**) qRT-PCR analysis of *fog-1* mRNA in WT and in *wdr-5.1;wdr-5.2.* (**C**) RNA purified from either whole L4 or adult worms grown at 25°C was analyzed by RT-PCR using *fog-3* specific primers and the products were visualized on a 1.2% agarose gel. *act-1* was used as an internal control.

The ability of *fog-3(RNAi)* to suppress the Mog phenotype of the *wdr-5.1;wdr-5.2* mutant indicates that these factors are required for the normal suppression of *fog-3* activity during the switch to oogenesis. WDR-5.1/5.2 may thus be required to promote oogenesis by inhibiting *fog-3* expression either directly or indirectly in a temperature sensitive manner, i.e. the Mog phenotype could be due to ectopic expression of *fog-3* in double mutant germ cells. To test this, we isolated gonads from both WT and *wdr-5.1/5.2* single and double mutant adult animals grown at 20 and 25°C and analyzed the expression levels of a number of sex-determination pathway genes. Of the 11 genes tested, only *fog-3* and *fog-1* showed significant regulation defects in the *wdr-5.1/5.2* double mutant gonads compared to controls (Figure 4A and B, Supplementary Figure S5). At 20°C, *fog-3* mRNA was increased 17.6-fold in *wdr-5.1;wdr-5.2* double mutants compared to that of WT. Notably, the double mutants show a mild penetrance (16%) of sterility associated with this level of *fog-3* misregulation (Table 1). No changes were found in single *wdr-5.1(ok1417*), *wdr-5.2(ok1444)*, or *set-2(tm1630)* mutants, again disconnecting the defects in *fog-3* regulation and H3K4 methylation, since H3K4 methylation defects are fully penetrant at 20°C in both of these mutants (14). At 25°C, however, *fog-3* mRNA levels increased dramatically in both the *wdr-5.1* single mutant and *wdr-5.1;wdr-5.2* double mutant by 48- and 77-fold, respectively (Figure 4A). Notably, *fog-3* mRNA levels correlated with the penetrance of the sterile phenotype, as 42% of the single and 100% of the double mutant animals were sterile (Table 1 and Figure 4A). The correlation, however, did not extend to the detailed phenotypes, since few of the sterile *wdr-5.1* single mutants were Mog (most were Emo), in contrast to the 88% Mog in the sterile *wdr-5.1;wdr-5.2* double mutants (Figure 2B), suggesting the ectopic expression of *fog-3* at levels observed in single *wdr-5.1* animals is insufficient for full manifestation of the Mog phenotype. We note that *fog-*3 is also subjected to post-transcriptional regulation (35). The apparent ‘threshold’ appearance of the Mog phenotype that we observe with the greatly increased *fog-3* dysregulation in the double mutants may represent a threshold titration of the post-transcriptional regulation processes.

*fog-3* expression is restricted to germ cells and required for spermatogenesis in larvae; its expression is repressed when oogenesis initiates in late larval/adult hermaphrodites (8). Consistent with this, RT-PCR analysis revealed that *fog-3* mRNA is present in L4 and absent in the germ cells of WT adults (Figure 4C). However, *fog-3* mRNA was detected in both L4 and adult germ cells in *wdr-5.1;wdr-5.2* double mutants (Figure 4C). Taken together, these results confirm that the germline sex determination defects we observe in *wdr-5.1;wdr-5.2* are linked to defects in repression of *fog-3* at the sperm/oocyte switch. They also suggest that *wdr-5.1* and *wdr-5.2* are significantly, but not completely redundant in their roles in *fog-3* regulation.

In contrast to *fog-3*, *fog-1* mRNA levels were not changed in *wdr-5.1;wdr-5.2* at 20°C. However, we observed a 6-fold increase in *fog-1* expression in *wdr-5.1;wdr-5.2* double mutants at 25°C compared with WT (Figure 4B). As mentioned above, RNAi of *fog-1* did not consistently revert the Mog phenotype of the double mutant, whereas *fog-3(RNAi)* strongly converted the Mog to Fog phenotype. The huge increase of *fog-3* (77-fold) in *wdr-5.1;wdr-5.2* is thus the predominant cause of the Mog phenotype.

### *fog-3* regulation is uncoupled from H3K4 methylation

WDR-5.1 and RBBP-5 are essential for global maintenance of both tri- and dimethylation of histone H3 on lysine 4 (H3K4me3 and H3K4me2, respectively) in early embryos and in germline stem cells (14). SET-2 is an H3K4 methyltransferase that is specifically required for the generation of H3K4me3 in these stages. Neither *set-2(tm1630)* nor *rbbp-5(tm3463)* showed obvious sex determination defects, although *rbbp-5* mutant animals, like *wdr-5.1* single mutants, display an oocyte endomitotic (Emo) phenotype at 25°C (14). The lack of Emo in the *set-2(tm1630)* germ cells could be due to the presence of H3K4me2 that is unaffected in this mutant (14). Therefore, with the exception of the low penetrance in *wdr-5.1* single mutants at 25°C, the Mog phenotype is not observed in any other Set/MLL component mutant we tested at any temperature. This further supports the conclusion that the defects in sperm/oocyte transition we observe in the *wdr-5* double mutants, which can be explained by *fog-3* derepression, is unlikely to be due to some indirect effect of defects in H3K4 methylation in the mutant germ cells.

**Figure 5.**
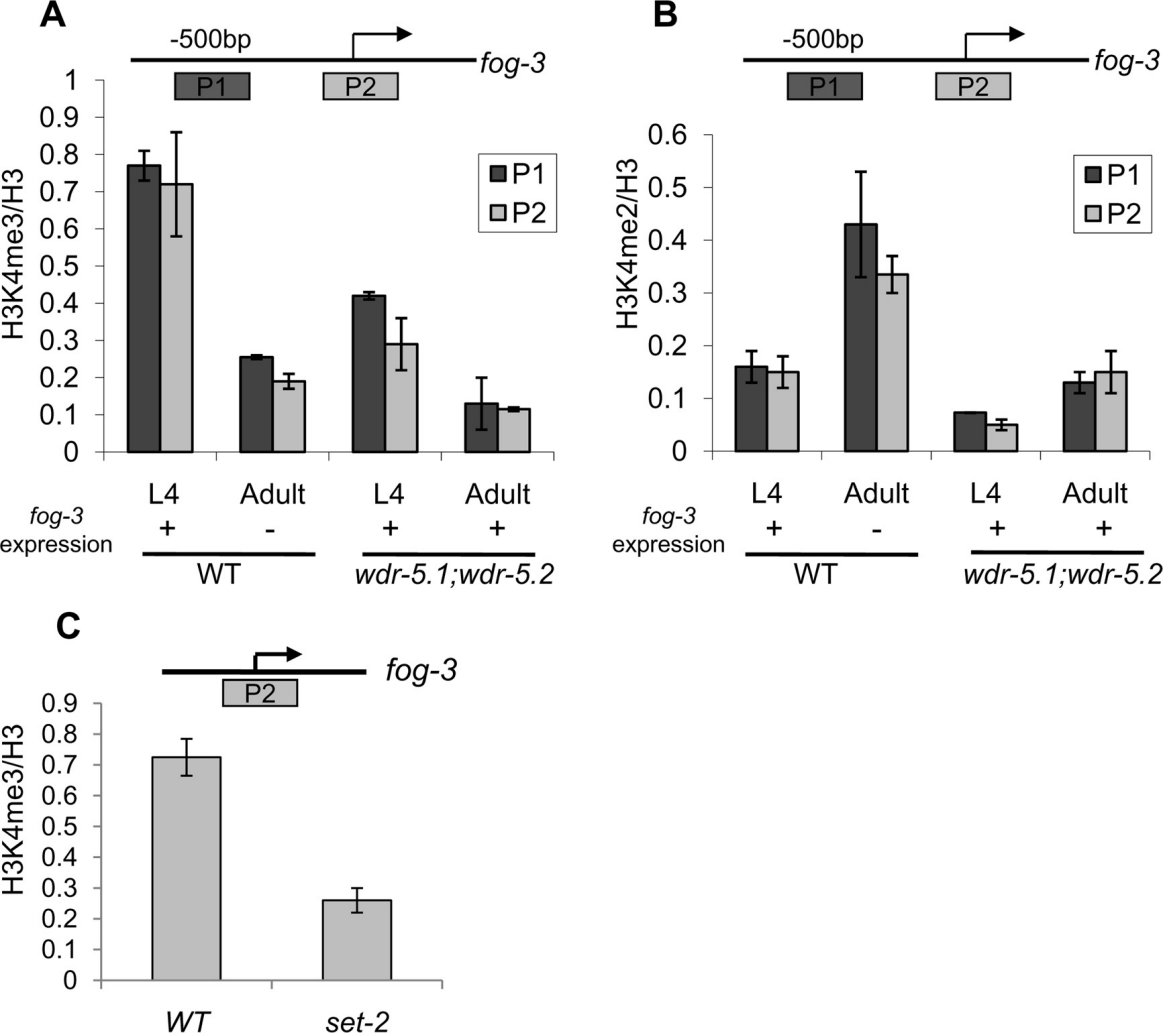
*set-2/wdr-5.1* dependent H3K4 methylation does not correlate with *fog-3* regulation. WT, *wdr-5.1;wdr-5.2* and *set-2* animals were synchronized as L1 larvae and grown at 25°C. L4 larval and adult stage (24 h post L4) animals were harvested for anti-H3K4me ChIP-qPCR analysis. H3K4 methylation levels at *fog-3* promoter were assayed using anti-H3K4me3, anti-H3K4me2 and anti-H3 antibodies. H3K4me2/3 signals were normalized to total histone H3 levels in parallel analyses. Arrows indicate transcription start sites. Error bars represent SEM based on three independent experiments. (**A**) ChIP assay of H3K4me3 at *fog-3* promoter regions; *fog-3* expression at different stages is from data in Figure 4B. (**B**) ChIP-qPCR assay for H3K4me2 levels in at the promoter regions of *fog-3*, with modified H3 normalized to total H3 ChIP. (**C**) H3K4me3 ChIP assay at the *fog-3* promoter in wild-type and *set-2* mutant L4 larvae.

To more directly test this, we next asked whether the regulation of *fog-3* by WDR-5.1/WDR-5.2 correlated with changes in H3K4 methylation at the *fog-3* locus. As previously mentioned, H3K4 methylation is most frequently associated with transcriptional competence and activity, thus increased *fog-3* expression correlating with a decrease in H3K4 methylation would itself be unusual. We used ChIP-qPCR to compare the levels of H3K4me2 and H3K4me3 at the *fog-3* promoter and transcriptional start site (TSS) regions in both WT and *wdr-5.1;wdr-5.2* L4 larvae and adults grown as 25°C. In WT animals, there is a decrease in H3K4me3 at the promoter and TSS in adults compared with L4's, which is predicted from transcriptional repression of *fog-3* in adults (Figure 5A). There is a corresponding increase in H3K4me2 in adults (Figure 5B), suggesting that the increased H3K4me3 in L4's may be due to transcription-dependent conversion of H3K4 di- to trimethylation. However, the methylation status of H3K4 at *fog-3* is not obligatorily linked to its expression. For example, H3K4me2 is retained at *fog-3* in WT adults despite its inactivation, which is consistent with a transcription-independent mode of H3K4me maintenance in adult germ cells that requires WDR-5.1 (14). In addition, in *wdr-5.1;wdr-5.2* mutant adults H3K4 methylation is lost from *fog-3* even though *fog-3* is ectopically expressed in these cells (Figure 5A and B). H3K4 methylation, by any mechanism, therefore does not always correlate with *fog-3* transcriptional activity. Furthermore, H3K4me3 at *fog-*3 requires SET-2 (Figure 5C). If dysregulation of H3K4me3 at *fog-3* was causative for ectopic *fog-3* regulation, the Mog phenotype would be observed in *set-2* mutants, and it was not. Taken together, these data strongly suggest that WDR5 can participate in the repression of *fog-3* in a mechanism that is independent of H3K4 methylation, and that defective H3K4 methylation does not correlate with defects in *fog-3* regulation.

### TRA-1 requires *wdr-5.1/wdr-5.2* for normal chromatin association in germ cells

The only known direct transcriptional regulator of *fog-3* is the TRA-1A isoform of the TRA-1/Gli repressor (8). TRA-1A binds to the *fog-3* promoter at the onset of oogenesis and this binding correlates with *fog-3* transcriptional repression (8,36). As *wdr-5.1* and/or *wdr-5.2* are required for the normal inhibition of *fog-3* (Figure 4), we hypothesized that WDR-5.1/WDR-5.2 and TRA-1 may work together or in parallel to inhibit *fog-3* expression. *tra-1* mRNA levels are unchanged in the *wdr-5.1;wdr-5.2* mutant compared to WT (Supplementary Figure S5), so it's unlikely that *wdr-5.1/wdr-5.2* indirectly inhibit *fog-3* transcription by affecting *tra-1* expression. We therefore tested whether TRA-1 protein abundance and/or localization were defective in the *wdr-5.1;wdr-5.2* double mutant.

**Figure 6.**
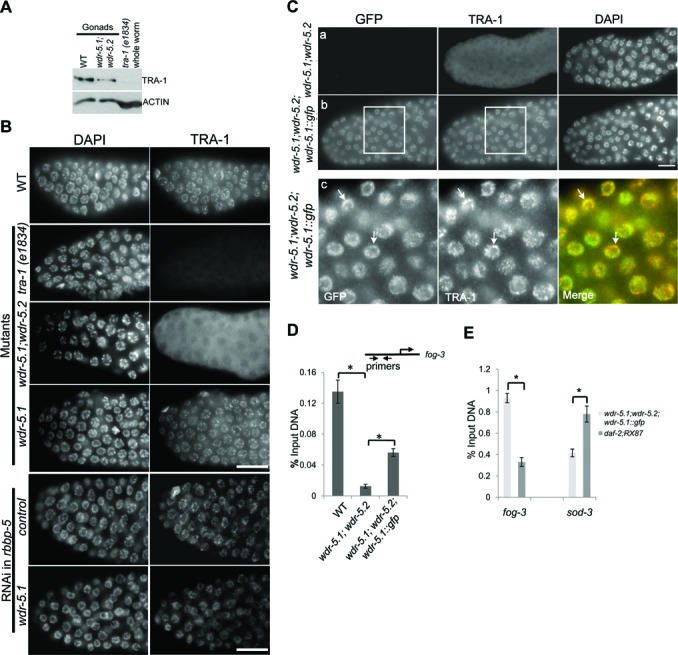
*wdr-5.1* and *wdr-5.2* are redundantly required for normal TRA-1 association with germline chromatin at 25°C. (**A**) Anti-TRA-1 western blots of lysates from WT and *wdr-5.1;wdr-5.2* dissected adult gonads grown at 25°C, compared with whole worm lysate of a *tra-1* mutant *(e1834)* as an antibody specificity control. The band marked as TRA-1 has the expected mobility of TRA-1A. (**B**) Anti-TRA-1 immunofluorescence analysis of dissected and whole-mount fixed gonads from WT or mutant animals grown at 25°C. In addition, *wdr-5.1* was knocked down by RNAi in *rbbp-5 (tm3463)* mutant animals. The F1 progeny of treated animals were grown at 25°C. Gonads dissected from F1 animals 24 h post L4 were stained with an antibody against TRA-1. Gonads were also counter-stained with DAPI. The exposure time is equal for each sample. Gonads are displayed with the distal (proliferative) region to the left. TRA-1 antibody signal is largely nuclear with a specific chromatin pattern in WT (top row) and this signal is absent in the *tra-1* mutant *(e1834)* (second row). The pattern is cytoplasmic in *wdr-5.1;wdr-5.2* gonads (third row) but is identical to WT in *wdr-5.*1 single mutants. Scale bar = 10 μm. (**C**) A *wdr-5.1::gfp* transgene restores TRA-1 nuclear and chromatin localization in *wdr-5.1;wdr-5.2* mutant. Adult *wdr-5.1;wdr-5.2* animals (a, without transgene, or b, carrying a *wdr-5.1::gfp* transgene) were grown at 25°C. Gonads were dissected and staining was performed as in (B). Anti-TRA-1 nuclear signal (middle panel) is restored with germline expression of the transgene (left panel). Panel C shows enlarged image of boxed area in (b). Arrows mark GFP and TRA-1 overlapped areas. Green: GFP; red: TRA-1. Scale bar = 10 μm. (**D**) Anti-TRA-1 ChIP-qPCR at *fog-3* promoter in WT, *wdr-5.1;wdr-5.2* double mutant, and double mutant animals rescued with a *wdr-5.1:gfp* transgene (*wdr-5.1;wdr-5.2; wdr-5.1::gfp*). Error bars represent SEM from two independent experiments. (**E**) WDR-5.1::GFP was associated with *fog-3* promoter at 25°C. GFP ChIP was done with GFP-TRAP_M in *wdr-5.1;wdr-5.2; wdr-5.1::gfp* and *daf-2;RX87* that carried GFP fused *daf-16*. Worms were grown at 25°C before collected for ChIP. Primers for *fog-3* are as shown in (D). Primers for *sod-3* are described in reference (14). The enrichment of GFP at *sod-3* served as a positive control for GFP ChIP. Error bars represent the SEM of the triplicated reactions in qPCR. Similar results were obtained from another independent experiment. *P < 0.05; Student's *t*-test.

To determine if TRA-1 protein levels were affected, adult hermaphrodite gonads were collected from WT and *wdr-5.1;wdr-5.2* double mutants grown at 25°C and lysates were analyzed by western blot using a TRA-1 specific antibody. As shown in Figure 6A, TRA-1A was readily detectable in lysates from both WT and *wdr-5.1;wdr-5.2* gonads, though there was a slight reduction in the mutant lysates relative to WT. The slight decrease may be due to a decrease in the germline stem cell pool that occurs in the *wdr-5.1* mutant (14). The predominant band detected by the antibody was MW = ∼100 kDa and is likely to be the processed TRA-1A isoform that is thought to play the major role in sperm repression (11).

We next probed for TRA-1A protein in adult hermaphrodite gonads by immunofluorescence microscopy, using an antibody against TRA-1 that recognizes all forms of TRA-1 (11). The immunofluorescence signal was specific to TRA-1 since no anti-TRA-1 staining was observed in the germ cells of a mutant strain carrying a null allele of *tra-1* (*e1834*; Figure 6B). In the distal germline stem cells of WT adult hermaphrodites, anti-TRA-1 staining was localized to the nucleus in a pattern consistent with association with DNA (Figure 6B). As expected, the nuclear TRA-1 antibody signal declined in germ cells as they progressed into early pachytene and became undetectable in later stages of meiosis (Supplementary Figure S6 and (36)). In animals raised at 20°C, the TRA-1 antibody pattern in *wdr-5.1;wdr-5.2* mutants was similar to WT (Supplementary Figure S6). However, in *wdr-5.1;wdr-5.2* double mutants grown at 25°C, TRA-1 distal germ cell nuclear staining was depleted with a concomitant increase in cytosolic antibody signal. Anti-TRA-1 staining patterns in single *wdr-5.1* mutants were not noticeably different than that of WT (Figure 6B). These results show that a defect in nuclear retention of TRA-1 correlates with the temperature-dependent germline sperm/oocyte switch defect in the *wdr-*5 double mutants. We then asked whether the TRA-1 retention defect was independent of H3K4 methylation activities. To address this, TRA-1 was stained in the gonads of *rbbp-5* and *rbbp-5* with *wdr-5.1* knockdown with RNAi. As shown in Figure 6B (bottom panel), the TRA-1 stainings were similar to WT, suggesting that the TRA-1 localization is independent of RBBP-5 and the H3K4 methylation activities.

To further test if the mislocalization of TRA-1 is dependent on WDR5, we introduced a *wdr-5.1::gfp* transgene into the *wdr-5.1;wdr-5.2* mutant strain. As shown in Figure 6C, WDR-5.1:GFP is expressed in distal germ cells and mainly localized on chromatin. Expression of this transgene also restored TRA-1's association with chromatin in adult germ cells (Figure 6C). Coinciding with this, fertility and normal sperm/oocyte switch was restored in the double mutant carrying the transgene at 25°C (Supplementary Figure S2). In addition, we observed extensive (but not complete) overlap between the anti-TRA-1 and anti-WDR-5.1::GFP patterns in the rescued germ cell nuclei (Figure 6C(c)). Thus, proper nuclear localization and DNA association of TRA-1 protein in the germline stem cells of adult hermaphrodites requires WDR5 function at 25°C. Furthermore, the overlapping immunofluorescence signals of anti-TRA-1 and WDR-5.1:GFP suggest that there is some level of interaction of these proteins in the genome, and in the absence of the interaction TRA-1 protein is not efficiently retained in the nucleus. We cannot rule out, however, that a post-translation modification that is dependent on WDR5 activity and required for TRA-1 association with chromatin is involved.

To specifically test whether the absence of *wdr-5.1/wdr-5.2* affects binding of TRA-1 at the *fog-3* promoter, we performed ChIP-qPCR using a TRA-1 specific antibody in WT and *wdr-5.1;wdr-5.2* mutant adult hermaphrodites grown at 25°C. We detected a strong enrichment of TRA-1 protein at the *fog-*3 in WT adult hermaphrodites. TRA-1 detection at the *fog-3* promoter was significantly decreased in *wdr-5.1;wdr-5.2* mutants grown at 25°C (Figure 6D). As with transgenic rescue of the Mog phenotype (Supplementary Figure S2), the association of TRA-1 with the *fog-3* promoter was partially restored in the double mutant by introduction of a transgene expressing *wdr-5.1::gfp* (Figure 6D), and the restoration was sufficient to rescue the Mog phenotype. These results confirm that the normal association of TRA-1A with chromatin and the *fog-3* promoter is dependent on WDR-5.1, and presumably redundantly dependent on WDR-5.2.

We then asked whether we could detect WDR-5.1 at the *fog-3* promoter. To address this, we used anti-GFP ChIP (GFP-TRAP M; see ‘Materials and Methods’ section) in the strain expressing WDR-5.1::GFP, followed by qPCR to detect binding to the same region of the *fog-*3 promoter where we observed TRA-1 binding. As a specificity control, we also performed anti-GFP ChIP-qPCR in a strain expressing DAF-16:*GFP* using primers specific for the *sod-3* promoter, a target of the DAF-16 transcription factor in the transgenic line used (33). We detected significant enrichment of WDR5:GFP at the *fog-3* promoter in the strain expressing WDR5:GFP, but no enrichment at the *sod-3* promoter in this line. In contrast, we only detected DAF-16::GFP enrichment at the *sod-3* promoter in the line expressing DAF-16:GFP and no anti-GFP enrichment at the *fog-3* promoter (Figure 6E). These data indicate that WDR-5.1 and TRA-1 are localized to the same region of the *fog-3* promoter in adult chromatin. Importantly, the anti-GFP ChIP-qPCR was performed in the transgene-rescued *wdr-5.1;wdr-5.2* double mutant strain at 25°C, verifying that the association can occur at the elevated temperature

Taken together, these results suggest that stable association of TRA-1 with germ cell chromatin and the *fog-3* promoter requires WDR-5.1 and/or WDR-5.2 at 25°C. The depletion of TRA-1 from the *fog-3* promoter in the absence of WDR5 directly leads to an ectopic expression of *fog-3* at 25°C that is sufficient to overcome all layers of *fog-*3 expression control in germ cells, thereby causing a Mog phenotype.

### TRA-1 mediated *mab-3* repression is also *wdr-5.1/wdr-5.2* dependent

In addition to *fog-3*, we also tested another known direct target of TRA-1, *mab-3* (10). The vitellogenin gene *vit-1* is expressed in intestinal cells only during female development because MAB-3 represses transcription of *vit-1* in males. *vit-1* expression is thus indirectly dependent on TRA-1 since TRA-1 inhibits *mab-3* expression in hermaphrodites (Supplementary Figure S7A). Therefore, defective TRA-1 repression in this genetic pathway is manifested in hermaphrodites as an ectopic *mab-3* expression leading to decreased expression of *vit-1*. To address whether *wdr-5.1/wdr-5.2* also participate in the regulation of these two genes, we performed qRT-PCR analysis using RNA purified from adult animals grown at 25°C. *mab-3* mRNA increased by 12-fold in *wdr-5.1;wdr-5.2* animals relative to WT (Supplementary Figure S7B). In agreement with this, *vit-1* mRNA levels were simultaneously reduced significantly in response to the elevated *mab-3* expression in the mutant (Supplementary Figure S7C). These data suggest that defective TRA-1-dependent repression is a common characteristic of *wdr-5.1/wdr-5.2* mutants.

## DISCUSSION

In this study, we provide evidence that *wdr-5.1* and *wdr-5.2* function redundantly in germline sex determination in *C. elegans*. Our data suggest that WDR-5.1 and WDR-5.2 are redundantly required for TRA-1 to bind stably to its target promoters at elevated temperatures. The WDR5 homologs are thus required for TRA-1 to stably repress transcription at its direct targets, including *fog-3* and *fog-1*, and provide an essential repressive function at these loci to ensure robust transcriptional control. *Caenorhabditis elegans* WDR-5.1, like its conserved yeast and mammalian counterparts, plays an essential role in histone H3K4 methylation and is a component of the equally conserved Set/MLL histone methyltransferase complex in worms (14,30). The role of WDR5 in TRA-1-mediated transcriptional repression, however, is clearly distinct from its role in H3K4 methylation. Our studies provide evidence that WDR5 can play important roles in gene regulation, including acting as a mediator of repression, and these functions are not always tied to its well-defined role in Set/MLL histone modifying complexes.

### *wdr-5.1* and *wdr-5.2* are redundantly required for TRA-1-mediated transcription repression

Mammalian WDR5 is a WD40 repeat protein and plays a central role in the assembly of the core Set/MLL complex and regulation of its HMT activity (16,17,26). In *C. elegans*, among the three WDR5 homologs, *wdr-5.1* alone is required for the normal maintenance of H3K4me3 and H3K4me2 in early embryos and the germline. Inactivation of *wdr-5.1* results in a dramatic loss of H3K4me2/3 from early embryonic and adult germline stem cell population, with defects in germline immortality (14,30,34). In contrast, both *wdr-5.2* and *wdr-5.3* are dispensable for H3K4 methylation and deletion of either gene does not produce any obvious germline phenotypes on its own.

WDR-5.1 and WDR-5.2 are largely redundant for regulation of normal switch from spermatogenesis to oogenesis. Interestingly, the redundancy appears to be at least partially due to an upregulation of *wdr-5.2* we have observed in the *wdr-5.1* mutant (Supplementary Figure S3). However, the functional redundancy is not complete, as there are detectable changes in *fog-3* expression in the *wdr-5.1* single mutant germ cells, indicating that WDR-5.2 activity only partially or poorly replaces WDR-5.1 function in this tissue. This is may be due to the X chromosome linkage of *wdr-5.2*, as X-linked genes are in general poorly expressed in the germline in *C. elegans* (37). The upregulation of *wdr-5.2* mRNA levels in *wdr-5.1* mutants, which itself is interesting but not yet explored, may be enough to compensate the Mog defects. However, the *wdr-5.2* upregulation does not seem to be able to complement either the H3K4 methylation defect or the Emo phenotype, which seems linked to the methylation defect. This indicates that these apparent paralogs have diverged in some aspects of their function. Consistent with this, a transgene expressing just WDR-5.1:GFP alone can rescue all aspects of WDR5 function, including the Emo and H3K4 methylation defects (14).

Both *fog-1* and *fog-3* are direct targets of TRA-1. We observed a dramatic increase in *fog-3* expression (77-fold) and a moderate upregulation in *fog-1* (6-fold). Knockdown of *fog-3* by RNAi can reverse the Mog phenotype to Fog. In contrast, *fog-1(RNAi)* could not rescue the Mog phenotype in *wdr-5.1;wdr-5.2* animals. Although we do not understand this discrepancy in reversion of the Mog phenotype by the different RNAi targets, RNAi knockdown of *fog-1* may have been insufficient to overcome the higher levels of ectopic expression of *fog-3* we observed in the double mutants.

Our data strongly suggest that the requirements for WDR5 in TRA-1-dependent regulation of germline sex determination are independent of the role of WDR5 in H3K4 methylation. First, *wdr-5.1* and *rbbp-5* are both non-redundantly required for global H3K4me2/3 maintenance in early embryos and adult germ cells (14). Disruption of either exhibits similar H3K4me2/3 pattern defects, indicating that a complex that includes both proteins, as is the case in yeast and mammals, is probably involved (17–19,38,39). Yet despite its completely penetrant H3K4 methylation defects in germ cells, *rbbp-5* mutants have no obvious defects in sperm/oocyte transition. This also makes it unlikely that a secondary TRA-1 interacting factor that is itself regulated by H3K4 methylation is involved. Moreover, while RNAi knockdown of either *wdr-5.1* or *rbbp-5* in *wdr-5.2* single mutants were able to abolish H3K4 methylation, only *wdr-5.1* knockdown yielded the Mog phenotype in *wdr-5.2* mutants (Figure 2C). Second, although WDR-5.2 is unable to compensate for the loss of WDR-5.1 activity in H3K4 methylation, it can compensate for WDR-5.1's role in sperm/oocyte transition. Third, H3K4 methylation at the *fog-*3 promoter does not strictly correlate with transcriptional activity at *fog-3*. This is consistent with our previous findings that H3K4 methylation in the distal germ cells, where TRA-1 repression occurs, is not always dependent on transcription (14). Thus, misregulation of H3K4 methylation in these cells may not correlate with transcriptional defects. Indeed, *set-2* is required for all H3K4me3 in the distal germ cells, and we have not detected any requirement for this H3K4 methyltransferase in sperm/oocyte transition. The most parsimonious explanation is that WDR5 has a separate function in TRA-1- mediated transcriptional repression that is not directly related to its function in the Set/MLL complex(es).

WDR5 has been shown to operate in complexes without HMT activities in other systems. For example, it has been suggested that a WDR5-containing complex interacts with NIF-1 to promote the expression of nuclear hormone receptor responsive genes (27). WDR5 is also found in human CHD8-containing ATP-dependent chromatin-remodeling complex and ATAC complex (28). Neither of these complexes is known to have HMT activities. Additionally, it has recently been shown that WDR5 is required for embryonic stem cell self-renewal, and interacts directly with the pluripotency factor Oct4 in these cells. Importantly, WDR5 interactions with Oct4 could be detected in complexes lacking other methyltransferase components (29). Our finding provides further evidence that WDR5 can play roles in transcriptional regulation that are separable from H3K4 methylation. It is thus important that such MLL-independent roles be considered when analyzing phenotypes that result from reduced WDR5 function in any system.

### WDR5 as a mediator for TRA-1 repression

The nematode sperm/oocyte decision is tightly controlled by a complex genetic pathway composed of a number of genes operating in a negative regulatory manner (Figure 3A). *tra-1* is the terminal transcription factor in this sex determination pathway. It has been shown that *tra-1* promotes female fate by repressing male-specific genes in all tissues (8,10,40). In the germline, *tra-1* facilitates oogenesis by inhibiting the expression of *fog-1* and *fog-3*, which are both required for initiation and maintenance of spermatogenesis (8,41).

TRA-1A plays a global role in sex determination, but how the activity of TRA-1 is regulated in the two sexes is not completely understood (8). It is reported that the overall levels of TRA-1 is similar in both sexes whereas nuclear TRA-1 levels are higher in hermaphrodites than in males, suggesting that nuclear localization plays a role in the regulation of TRA-1 activity (12). However, the opposite conclusion was made in another study which found that TRA-1 was primarily localized in the nuclei in both sexes while the hermaphrodites have higher protein levels of TRA-1 (11). Although our results do not differentiate between these models, it is clear from our studies that defective nuclear localization of TRA-1 largely correlates with defective TRA-1-mediated transcriptional repression. Importantly, we observed few somatic defects that are normally associated with defective *tra-1* function. For example, *tra-1* loss-of-function mutants exhibit significant somatic masculinization, but this phenotype was not apparent in the *wdr-5* double mutants at any temperature. We did see male tail defects in the double mutants (not shown), which is likely attributable to the observed misregulation of *mab-3*, but this seems to be separable from defective sex determination since the hermaphrodites exhibiting *mab-3* derepression did not have masculinized tails.

How does WDR5 contribute to TRA-1 repression of its targets? Our results indicate that it is required for efficient and stable localization of TRA-1 at the *fog-*3 promoter and the subsequent transcriptional repression of *fog-3* by TRA-1. It is interesting that although the phenotypic consequences of WDR5 defects are only evident at elevated temperatures, significant transcriptional derepression of *fog-3* is also observed at 20°C (Figure 4A). This indicates that while stable promoter association and repression by TRA-1 requires WDR5 at all temperatures, other modes of regulation of TRA-1 targets (e.g. post-transcriptional regulation) may help suppress the Mog phenotype at the lower temperatures. At elevated temperatures, the association of TRA-1 at its targets is further weakened in the absence of WDR5, and the subsequent more robust ectopic expression may override secondary regulation. The stabilization of TRA-1's association with target loci may be mediated by WDR5's interactions with chromatin. WDR5 interacts with histone H3, which may help stabilize TRA-1 at a target promoter and/or help with the repressor function of TRA-1. For example, WDR5 could bridge an interaction between TRA-1 and an adjacent nucleosome, stabilizing a nucleosome at or near the TSS to interfere with transcription initiation. The MLL histone methyltransferase's association with WDR5 is via the same domain with which WDR5 interacts with histone H3 (26), suggesting that WDR5's binding to histone H3 can compete with its association with the MLL complex. Based on our observation that both WDR5 and TRA-1 bind to the same region of *fog-3* promoter in *wdr-5.1*, *wdr-5.2* adult (Figure 6), it is interesting to speculate that WDR5, recruited to loci as a component of the Set/MLL complex, might be retained at a locus independently of the complex via its interactions with histone H3. Competition for WDR5 binding between MLL and histone H3 at these sites could lead to disruption of the MLL complex, thereby limiting and/or regulating H3K4 methylation, a modification that appears to have little spreading in genomes analyzed. The retained WDR5 could also provide a ‘landing pad’ for other proteins required for further regulation of the locus, such as the role described here in TRA-1-dependent repression.

It will be interesting to identify how WDR5's interactions with TRA-1, and perhaps other transcriptional regulators, fit together with its interactions with histone H3. Furthermore, although *C. elegans* does not have a known Hedgehog-signaling pathway, our results suggest a paradigm for Ci/Gli repressor-mediated transcriptional regulation that may be relevant for those organisms in which this pathway exists.

## SUPPLEMENTARY DATA

Supplementary Data are available at NAR online.

SUPPLEMENTARY DATA
