# Supplementary material for: A role for WDR5 in TRA-1/Gli mediated transcriptional control of the sperm/oocyte switch in C. elegans
Source: Nucleic Acids Res. 2014 Mar 20;42(9):5567–81. doi: 10.1093/nar/gku221 (PMC4027197; doi:10.1093/nar/gku221)
Supplement: SUPPLEMENTARY DATA [file supp_42_9_5567__index.html]

A role for WDR5 in TRA-1/Gli mediated transcriptional control of the sperm/oocyte switch in C. elegans — SUPPLEMENTARY DATA 

# A role for WDR5 in TRA-1/Gli mediated transcriptional control of the sperm/oocyte switch in *C. elegans*

## SUPPLEMENTARY DATA

**Files in this Data Supplement:**

- SUPPLEMENTARY DATA
- SUPPLEMENTARY DATA
