# Supplementary material for: A role for WDR5 in TRA-1/Gli mediated transcriptional control of the sperm/oocyte switch in C. elegans
Source: Nucleic Acids Res. 2014 Mar 20;42(9):5567–81. doi: 10.1093/nar/gku221 (PMC4027197; doi:10.1093/nar/gku221)
Supplement: SUPPLEMENTARY DATA [file supp_gku221_nar-01555-x-2013-File008.pdf]

A

```
hWDR5           -------VKPNYALKFTLAG-HTKAVSSVKFSPNGEWLASSSADKLIKIWGAYDGKFEKT 52
CeWDR-5.1       -----ASGSANYKLMCTLEG-HTKSISSAKFSPCGKYLGTSSADKTVKIWNMDHMICERT 54
CeWDR-5.2       PNPSAANLWPTYKLVAEIPNAHKKSISGIKFSPDGRYMGSGSADCSIKIWRMD-FVYEKT 59
CeWDR-5.3       ---------GEFSLVKTISG-HTKSVSVIKFSYCGKYLGTGSADKQIKVWNTVDMTYLQT 50
                           : *   : . *.*::*  ***  *.::.:.***  :*:*        :*

hWDR5           ISGHKLGISDVAWSSDSNLLVSASDDKTLKIWDVSSGKCLKTLKGHSNYVFCCNFNPQSN 112
CeWDR-5.1       LTGHKLGVNDIAWSSDSRCVVSASDDKTLKIFEIVTSRMTKTLKGHNNYVFCCNFNPQSS 114
CeWDR-5.2       LMGHRLGINEFSWSSDSKLIVSCSDDKLVKVFDVSSGRCVKTLKGHTNYVFCCCFNPSGT 119
CeWDR-5.3       LASHQLGINDFSWSSNSQFIASASDDTTVKIFDVISGACLRTMRGHTNYVFCCSFNPQSS 110
                : .*:**:.:.:***:*. :.*.***. :*:::: :.   :*::**.****** ***...

hWDR5           LIVSGSFDESVRIWDVKTGKCLKTLPAHSDPVSAVHFNRDGSLIVSSSYDGLCRIWDTAS 172
CeWDR-5.1       LVVSGSFDESVRIWDVKTGMCIKTLPAHSDPVSAVSFNRDGSLIASGSYDGLVRIWDTAN 174
CeWDR-5.2       LIASGSFDETIRIWCARNGNTIFSIPGHEDPVSSVCFNRDGAYLASGSYDGIVRIWDSTT 179
CeWDR-5.3       LIASAGFDETVRVWDFKTGLCVKCIPAHSDPITSISYNHDGNTMATSSYDGCIRVWDAAS 170
                *:.*..***::*:*  :.*  :  :*.*.**:::: :*:**  :.:.****  *:**::.

hWDR5           GQCLKTLIDDDNPPVSFVKFSPNGKYILAATLDNTLKLWDYSKGKCLKTYTGHKNEKYCI 232
CeWDR-5.1       GQCIKTLVDDENPPVAFVKFSPNGKYILASNLDSTLKLWDFSKGKTLKQYTGHENSKYCI 234
CeWDR-5.2       GTCVKTLIDEEHPPITHVKFSPNGKYILASNLNNTLKLWDYQKLRVLKEYTGHENSKYCV 239
CeWDR-5.3       GSCLKTLVDTDHAPVTFVCFSPNGKYLLSAQLDSSLKLWDPKKAKPLKYYNGHKNKKYCL 230
                * *:***:* ::.*::.* *******:*:: *:.:***** .* : ** *.**:*.***:

hWDR5           FANFSVTGGKWIVSGSEDNLVYIWNLQTKEIVQKLQGHTDVVISTACHPTENIIASAALE 292
CeWDR-5.1       FANFSVTGGKWIISGSEDCKIYIWNLQTREIVQCLEGHTQPVLASDCHPVQNIIASGALE 294
CeWDR-5.2       AANFSVTGGKWIVSGSEDHKVYIWNLQTREILQTLDGHNTAVMCTDCHPGQNIIASAALE 299
CeWDR-5.3       FANMSVPLGKHIISGSEDGRILVWSIQTKQIVQILEGHTTPVLATDSHPTLNIIASGGLE 290
                 **:**. ** *:*****  :  :*.:**::*:* *:**.  *:.: .**  *****..**

hWDR5           NDKTIKLWK 301
CeWDR-5.1       PDNKIHIWR 303
CeWDR-5.2       PDMRIKIWR 308
CeWDR-5.3       PDNVIRIW- 298
                 *  *::*
```

B

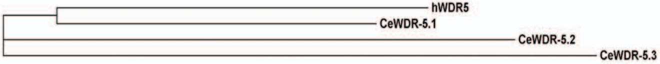

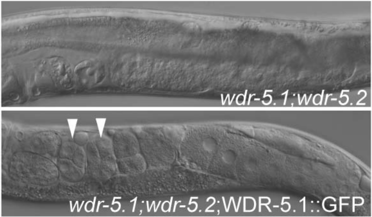
wdr-5.1;wdr-5.2
wdr-5.1;wdr-5.2;WDR-5.1::GFP

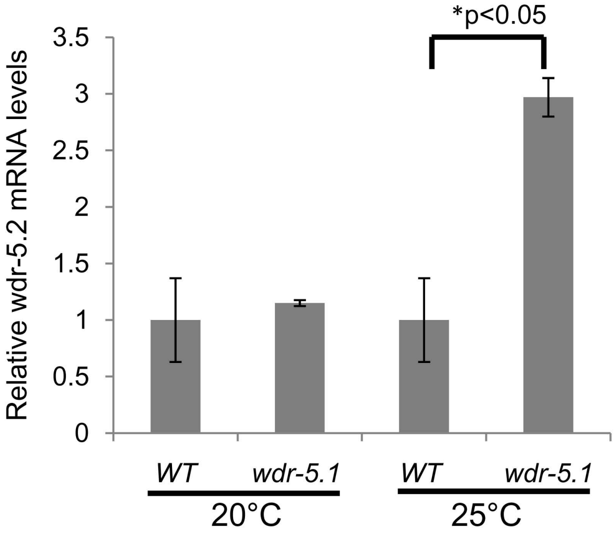

*p<0.05
Relative wdr-5.2 mRNA levels
3.5
3
2.5
2
1.5
1
0.5
0
WT
wdr-5.1
WT
wdr-5.1
20°C
25°C

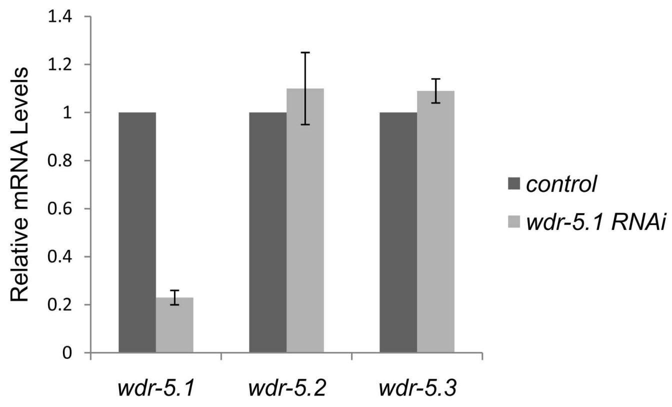
1.4
1.2
1
0.8
0.6
0.4
0.2
0
Relative mRNA Levels
control
wdr-5.1 RNAi
wdr-5.1
wdr-5.2
wdr-5.3

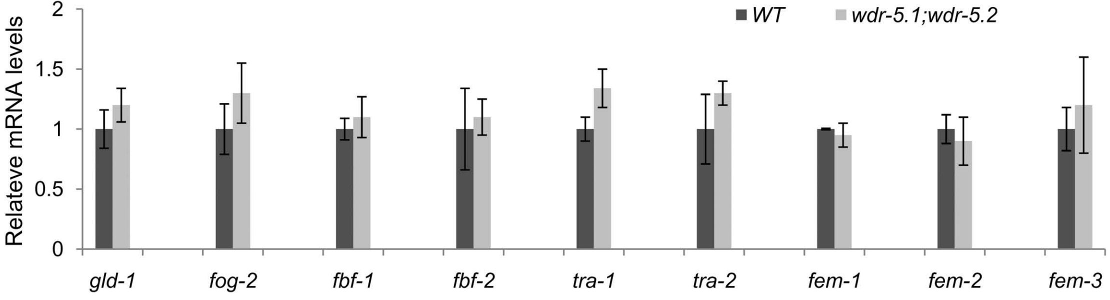

Relateve mRNA levels
0
0.5
1
1.5
2
WT
wdr-5.1;wdr-5.2
gld-1
fog-2
fbf-1
fbf-2
tra-1
tra-2
fem-1
fem-2
fem-3

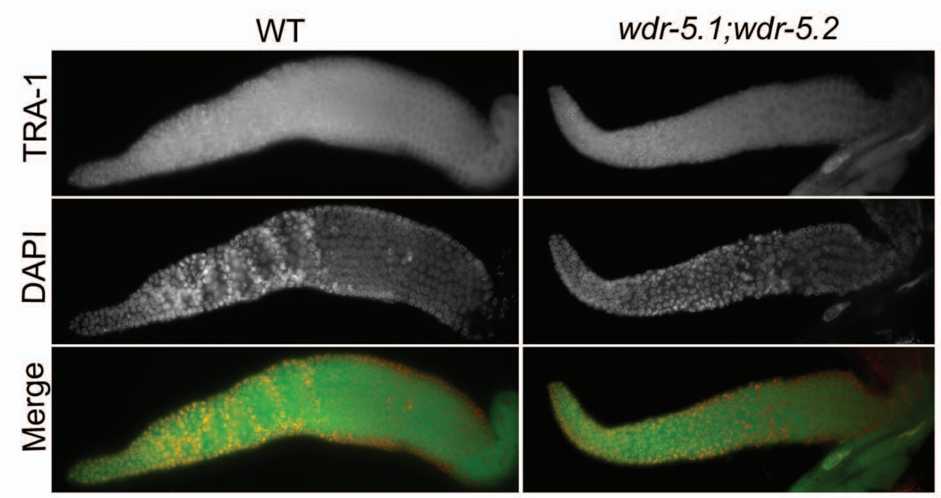
WT
wdr-5.1;wdr-5.2
TRA-1
DAPI
Merge

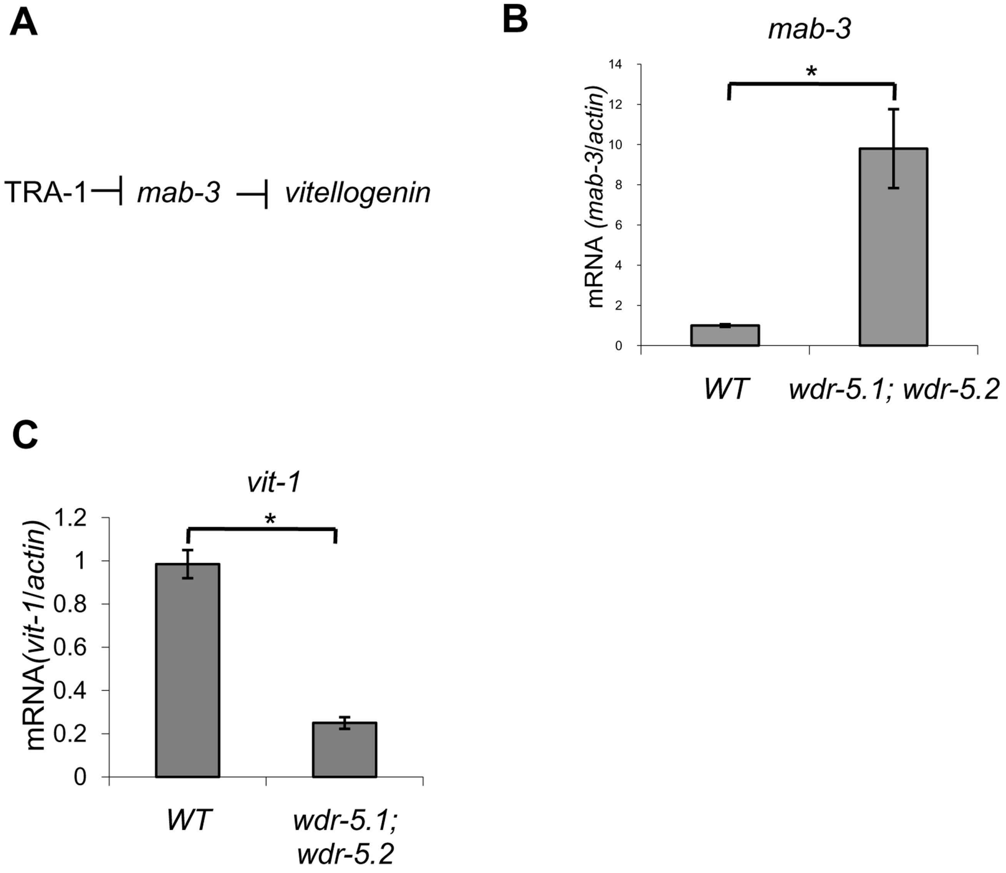
A
TRA-1 ⊣ mab-3 ⊣ vitellogenin
B
mab-3
mRNA (mab-3/actin)
0
2
4
6
8
10
12
14
*
WT
wdr-5.1; wdr-5.2
C
vit-1
mRNA(vit-1/actin)
0
0.2
0.4
0.6
0.8
1
1.2
*
WT
wdr-5.1;
wdr-5.2
