# Supplementary material for: A role for WDR5 in TRA-1/Gli mediated transcriptional control of the sperm/oocyte switch in C. elegans
Source: Nucleic Acids Res. 2014 Mar 20;42(9):5567–81. doi: 10.1093/nar/gku221 (PMC4027197; doi:10.1093/nar/gku221)
Supplement: SUPPLEMENTARY DATA [file supp_gku221_nar-01555-x-2013-File009.docx]

**Supplemental Table S1. Primers used**

**Supplemental Figure S1. Sequence alignment of WD40 repeat of human and worm WDR-5 proteins**

The alignment was generated using ClustalW2. An * (asterisk) indicates positions which have a single, fully conserved residue; A : (colon) indicates conservation between groups of strongly similar properties; A . (period) indicates conservation between groups of weakly similar properties. Colors indicate the properties of the amino acids.

**Supplemental Figure S2. *wdr-5.1;wdr-5.2* sterility is rescued by a *wdr-5.1* transgene**

Differential interference contrast (DIC) micrographs of the gonad regions of adult *wdr-5.1;wdr-5.2* and *wdr-5.1;wdr-5.2;wdr-5.1:gfp* hermaphrodites grown at 25°C. Arrow heads mark embryos.

**Supplemental Figure S3. *wdr-5.2* is upregulated in *wdr-5.1* at 25°C**

RNA was purified from dissected gonads and RT-PCR was done as in Figure 3. The actin gene *act-1* was used as an internal control. Signals were normalized to WT, which was artificially set as 1. Error bars represent SEM from 2 independent experiments.

**Supplemental Figure S4. RNAi knockdown of *wdr-5.1* is specific and does not affect the mRNA levels of *wdr-5.2* and *wdr-5.3***

*wdr-5.1(RNAi)* was performed at 20°C . RNA was purified from 100 adult worms. The actin gene *act-1* was used as an internal control. Signals were normalized to the RNA levels of worms from RNAi control, which was artificially set as 1. Results are from two independent experiments. Error bars represent SEM.

**Supplemental Figure S5. qRT-PCR analysis of a panel of sex determination genes in *wdr-5.1;wdr-5.2* mutants compared to wild type**

RNA was purified from dissected gonads and qRT-PCR was done as in Figure 3. The actin gene *act-1* was used as an internal control. Signals were normalized to WT, which was artificially set to equal 1.0. Results are from two independent experiments, each performed in triplicate. Error bars represent SEM.

**Supplemental Figure S6. TRA-1 localization is normal in *wdr-5.1;wdr-5.2* mutant at 20°C.** Adult animals were grown at 20°C; gonads were dissected and immunofluorescence was performed as in Figure 6. DAPI is shown in red and TRA-1 in green. TRA-1 is largely nuclear localized in the distal cells of wild-type gonads, and this nuclear localization declines in proximal region and is replaced by a cytoplasmic enrichment. This wild-type pattern also occurs in *wdr-5.1;wdr-5.2* adults at this temperature.

**Supplemental Figure S7. *wdr-5.1;wdr-5.*2 are required for TRA-1-dependent *mab-3* repression at 25°C**

qRT-PCR analyses of *mab-3* and its target, *vit-1,* in wild-type and *wdr-5.1;wdr-5.2* animals grown at 25°C. *actin-1* was used as an internal control. Results were normalized to wild-type which was artificially set as 1. Error bars represent SEM based on 2 independent experiments. (A) Genetic interactions controlling *vitellogenin* expression. Barred lines indicate negative regulations. (B) *mab-3* mRNA is upregulated in *wdr-5.1;wdr-5.2.* *p<0.05; Student’s t test. (C) *vit-1* mRNA is reduced in *wdr-5.1;wdr-5.2*, presumably due to the ectopic *mab-3* expression shown in (B)*.* *p<0.05; Student’s t test.

**Supplemental Table S1**

| qRT-PCR primers | *fog-3-F* | TTTATCGGAGAACGGAATCG |
| --- | --- | --- |
|  | *fog-3-R* | ACGTACTCGGGGACTGATTG |
|  | *act-F* | TGCTGATCGTATGCAGAAGG |
|  | *act-R* | TAGATCCTCCGATCCAGACG |
|  | *mab-3-F* | CCCGAGATGGTAAAGAACCA |
|  | *mab-3-R* | TGGACTTGCTGATGTTCCAA |
|  | *wdr-5.1-RT-F* | TTGTGGGATTTCTCGAAAGG |
|  | *wdr-5.1-RT-R* | CCATTTTCCACCGGTAACAG |
|  | *wdr-5.2-RT-F* | CGGGATCCTACGATGGAATA |
|  | *wdr-5.2-RT-R* | GATTGGAGGATGCTCTTCGT |
|  | *wdr-5.3-RT-F* | GGTCCCACTTGGAAAGCATA |
|  | *wdr-5.3-RT-R* | GTTGCCAAAACGGGAGTAGT |
|  | *vit-1-F* | CACAGTTCTGAAGCCAGACG |
|  | *vit-1-R* | AATAAGCGACGCAAGCAACT |
| ChIP primers | *fog-3-F-promoter* | gaagctcgcactttcgtttt |
|  | *fog-3-R-promoter* | cgcggacttcggtatacatt |
|  | *fog-3-F2* | CAATGTATTCTCCATTGGCG |
|  | *fog-3-R2* | TTTGAGCAGATGACGGCTTG |
| Genotype primers | *wdr-5.1-F* | ATTGTGTGTTCGCTGTGCAT |
|  | *wdr-5.1-R* | TCTGGAGATTGGAGATGTAG |
|  | *wdr-5.1-R2* | GAACGGATTCATCGAATGATCCGG |
|  | *wdr-5.2-F* | TCTGGCAGTGTGCAAATGAT |
|  | *wdr-5.2-R* | TCGAGTCGCTGCTCCATGAG |
|  | *wdr-5.2-R2* | TGGAGCAACTTGAGCAAAGATATG |
